# Cow’s Milk Substitutes for Children: Nutritional Aspects of Milk from Different Mammalian Species, Special Formula and Plant-Based Beverages

**DOI:** 10.3390/nu11081739

**Published:** 2019-07-27

**Authors:** Elvira Verduci, Sofia D’Elios, Lucia Cerrato, Pasquale Comberiati, Mauro Calvani, Samuele Palazzo, Alberto Martelli, Massimo Landi, Thulja Trikamjee, Diego G Peroni

**Affiliations:** 1 Department of Pediatrics, San Paolo Hospital, 20142 Milan, Italy; 2 Department of Health Science, University of Milan, 20142 Milan, Italy; 3 Department of Clinical and Experimental Medicine, Section of Paediatrics, University of Pisa, 56126 Pisa, Italy; 4 Department of Clinical Immunology and Allergology, I.M. Sechenov First Moscow State Medical University, 119991 Moscow, Russia; 5 Department of Pediatrics, S. Camillo-Forlanini Hospital, 00152 Rome, Italy; 6 Department of Pediatrics, Garbagnate Milanese, 20024 Milan, Italy; 7 National Pediatric Health Care System, 10135 Turin, Italy; 8 Allergy &Immunology Unit, University of Cape Town Lung Institute, 7700 Cape Town, South Africa

**Keywords:** allergy, children, cow’s milk allergy, goat’s milk, non-dairy milk, plant-based beverages, plant-based milk, milk formula, nutrition

## Abstract

Cow’s milk and dairy are commonly consumed foods in the human diet and contribute to maintaining a healthy nutritional state, providing unique sources of energy, calcium, protein, and vitamins, especially during early childhood. Milk formula is usually made from cow’s milk and represents the first food introduced into an infant’s diet when breastfeeding is either not possible or insufficient to cover nutritional needs. Very recently, increased awareness of cow’s milk protein allergy and intolerance, and higher preference to vegan dietary habits have influenced parents towards frequently choosing cows’ milk substitutes for children, comprising other mammalian milk types and plant-based milk beverages. However, many of these milk alternatives do not necessarily address the nutritional requirements of infants and children. There is a strong need to promote awareness about qualitative and quantitative nutritional compositions of different milk formulas, in order to guide parents and medical providers selecting the best option for children. In this article, we sought to review the different compositions in terms of macronutrients and micronutrients of milk from different mammalian species, including special milk formulas indicated for cow’s milk allergy, and of plant-based milk alternatives.

## 1. Introduction

Cow’s milk (CM) and dairy products represent basic foods for human nutrition and development, being unique sources of energy, nutrients, calcium, proteins, and vitamins. CM formula is usually the first food introduced into an infants’ diet when breastfeeding is either not possible or insufficient to cover nutritional needs. Of note, CM and dairy continue to be an essential part of nutrition throughout life, being recommended as an essential food group for daily consumption. The average composition of CM constitutes 3.5% protein (80% caseins, 20% serum proteins), 3–4% lipid (triglycerides), 4.6% carbohydrate (lactose), 1% mineral salts (calcium, phosphorus, potassium, magnesium, sodium), vitamins (especially B1, B2, B6, retinol, carotenes, tocopherol) and 88% water [1]. 

Although CM is the most widespread type of milk, accounting for 83% of global production, the use of milk from other animal species has increased in recent years. Buffalo milk accounts for 13% of global production of milk and dairy products, while the contribution of goat’s (2.3%), sheep’s (1.4%) and camel’s (0.3%) milk is limited. Other species, such as reindeer, yak, elk, musk ox, llama, and alpaca account for 0.2% of the world milk market. Very recently, increased awareness of cow’s milk protein allergy (CMPA) and intolerance, and higher prevalence of vegan dietary habits have influenced parents towards frequently choosing cows’ milk substitutes for children, comprising other mammalian milk alternatives and plant-based milk beverages [2]. However, many of these milk alternatives do not necessarily address the nutritional requirements of infants and children.

The nutritional composition of milk alternatives to CM has received little attention from scientific research. The lack of such information is a critical unmet need, because, these milk alternatives have the potential to contribute to food security, health, and nutrition of a population [1]. 

These milk alternatives vary in their composition of macro and micronutrients [3]. In terms of macronutrients, one can distinguish milk rich in proteins, fats, and lactose, such as reindeer and elk, from those characterized by a low protein, fat and high lactose content, such as mare and donkey milk (Table 1). Lactose is the main carbohydrate of milk, and it is involved in intestinal absorption of calcium, magnesium, and phosphorus, in the use of vitamin D, in brain development and is a source of energy. As much as 30% of CM energy, about 40% of breast milk energy and 53–66% equine milk energy comes from lactose [1].

In this review, we sought to review the different compositions in terms of macronutrients and micronutrients of milk from different mammalian species, including special milk formulas indicated for CMPA, and of plant-based milk alternatives.

## 2. Mammalian Milk Alternatives

### 2.1. Buffalo Milk

Buffalo milk contains more than twice the amount of CM fat (7.5 g/100g vs. 3.3 g/100g), resulting in higher energy content; the percentage of saturated fats (65–75 g/100g of total fatty acids {FA}) is comparable to that of CM. The high-fat content makes buffalo milk particularly suitable for the production of dairy products (e.g., 1 kg of butter is produced with 10 kg of buffalo milk, compared to 14 kg of CM). Buffalo milk has protein levels slightly higher than those of CM, while the amount of lactose is similar [1].

### 2.2. Goat’s Milk

Goat’s milk has a nutritional composition very similar to CM in terms of saturated fats and quantity of trans-FA. Goat’s milk is abundant in short-chain, and medium-chain FA (6–10 C atoms), present in quantities even twice as much as CM; these FA are metabolized differently from those with a long chain and are rapidly available sources of energy. Furthermore, the reduced size of fat globules makes goat’s milk easier to digest. The typical “goat flavor” seems to be linked to the presence of branched-chain fatty acids with fewer than 11 C atoms.

As far as the carbohydrate content, goat’s milk contains changing quantities of lactose, depending on the diet of the animal, and high levels of oligosaccharides (the content of sialic acid is four times higher than that of CM). Goat milk protein quantity is comparable to that of CM.

Regarding micronutrients composition, goat’s milk has higher retinol content, lower levels of vitamin B12, significantly lower levels of folate, and higher levels of free amino acids (especially taurine) compared to CM [1].

### 2.3. Sheep’s Milk

The average size of fat globules is lower in sheep’s milk than in CM, which makes it easier to digest. It has high levels of protein (5.6 g/100 g) and lipids (6.4 g/100 g); among the most common species, only buffalo milk has more fat content than sheep. The FA profile of sheep’s milk is quite similar to that of goat’s milk: five FA represent more than 75% of fat, and the content of saturated FA (65–75 g/100 g of total FA) is comparable to that of CM, buffalo’milk, and goat’s milk. Sheep’s milk also contains more lactose than human milk, CM, and goat milk.

Furthermore, sheep’s milk contains lower levels of sodium and potassium than human and CM, whereas most of the other minerals are present in higher quantities. Finally, sheep’s milk has a higher level of retinol than goat’s and CM. Similar to goat’s milk, sheep’s milk also contains the amino acid taurine [1].

### 2.4. Milk from Horse and Donkey

Mares and donkeys produce a similar type of milk, with no significant differences in the proteins, lipids, lactose and mineral salt contents. The composition of horse and mare milks is more similar to that of breast milk due to the high levels of lactose, the low levels of proteins, caseins (40–45% of total proteins), and mineral salts. However, it should be noted that the fat content of horse and donkey is significantly lower than breast milk with consequent lower energy content. These milk alternatives have a lower protein and fat content than CM with high levels of polyunsaturated fatty acids (PUFA) and low levels of saturated FA. They contain alpha-linolenic acid (ALA, Omega 3 series) and linoleic acid (LA, omega 6 series), which are essential FA and precursors of docosahexaenoic acid (DHA) and arachidonic acid (AA), respectively. The high levels of these precursor FA are likely attributable to the fact that mares and donkeys are monogastric animals so that FA are not hydrogenated before absorption, as happens in ruminants, causing them to be transformed into saturated FA [1].

The differences between mare and donkey milk are the following:

- Donkey’s milk does not contain trans-FA and conjugated linoleic (CLA);
- Mare’s milk contains trans-FA and CLA in negligible quantities;
- Mare’s milk contains up to 15 mg of acid ascorbic/100 g, much more than CM.

Trans-FA are naturally found in foods derived from ruminants (milk and dairy products). They can be unsaturated and polyunsaturated with at least one double bond in trans-configuration. The vaccine acid (C18: 1, trans-11) is one of the most abundant trans-FA present in CM and breast milk (according to the mother’s diet); it is the intermediate precursor of conjugated linoleic acid (C18: 2, cis-9, trans-11). These natural trans-FA have different properties compared to trans-FA originating from partially hydrogenated vegetable oils and have critical immune-regulatory functions.

### 2.5. Milk from Dromedary and Camel

The dromedary and the camel have an important social and nutritional role in the arid and semi-arid areas as a source of milk for the population; they are called semi-ruminants because they have a stomach with three compartments (instead of 4) with functional properties similar to those of ruminants.

The dromedary and camel milk alternatives have similar amounts of lactose, but different quantities of lipid (higher in camel milk). Both contain 1–2 g of ALA and LA/100 g of total FA. The dromedary milk has a composition very similar to CM, with a slightly lower saturated content (60 g/100 g total FA) and higher monounsaturated fatty acids (MUFA) content. Camel milk has a lower saturated fatty acid content than cow’s (50 g/100 g total FA). The most important feature of camel and dromedary milk alternatives concerns the protein fraction: the β-lactoglobulin levels are not measurable (similar to breast milk); the most common serum protein is α-lactalbumin; the main casein is β-casein (similar to mother’s milk). These features give these two types of milk greater digestibility and a lower incidence of allergies compared to CM.

Furthermore, both milk alternatives have higher amounts of bioactive and antimicrobial substances (lysozyme, lactoferrin, immunoglobulins) than CM and buffalo’s milk. Vitamin C levels in dromedary milk are usually between 2.5 mg/100 g to 18.4 mg/100 g, depending on the breed. However, it must be kept in mind that camel and dromedary vitamin C are more sensitive to the heat than that found in CM and can be reduced by about 27% when milk is pasteurized [1].

### 2.6. Musk Ox Milk

The musk ox is an arctic mammal, belonging to the subfamily Caprinae, such as goat and sheep. Limited data is available on the composition of this milk. From what is known so far, musk ox milk contains more protein and lipid (5.4 g/100 g of lipid, but not a high quantity for an Arctic animal) compared to CM. The amount of lactose and water is lower, while the mineral salts content is more than double the CM (1.6 g/100 g vs. 0.7 g/100 g) [1].

### 2.7. Yak Milk

Unique bovine bred in the mountains of China, Mongolia, Russia, Nepal, and Uzbekistan. Different plants in these regions produce milk from Yak powder for domestic use. The composition of yak milk is very similar to that of buffalo milk, from which it differs only in total protein content. As in buffalo milk, the fat content of yak milk is much higher than that of CM. The predominant FA in yak milk are the same as in cow and buffalo milk, and only a small amount of PUFA is reported (2 g/100 g of total FA). Saturated fats represent about 65 g/100 g of total FA. The short-chain FA content is low, and small amounts of CLA have also been reported. Besides, in yak’s milk, compared to CM, there is an almost double quantity of β-lactoglobulin, and the levels of lactoferrin are 2–6 times higher [1].

### 2.8. Mithun Milk

Mithun is mainly found in the hilly regions of India, Myanmar, Bangladesh, where it plays an essential role in the economic, social, and cultural life of local populations. Few studies are available on the composition of this milk. It contains higher content in total fat (8.9 g/100 g) and total protein (6.5 g/100 g) than CM (3.3 g fat and 3.3 g protein/100 g milk); this is due to the tonnage of this species and its low yield in milk production [1].

### 2.9. Milk from Llama and Alpaca 

Little information is available on llama and alpaca milk alternatives. Both species represent a nutritional and economic resource that has not always been exploited by people living in the mountainous areas of South America.

Alpaca milk is more abundant in protein and mineral salts than the milk of other camelids and CM; no studies on its lipid composition are available. The milk of llama does not contain measurable levels of β-lactoglobulin. Concerning the lipid profile, it has proportions of saturated, monounsaturated and polyunsaturated fats comparable to those of CM; contains trans-FA (3 g/100 g total FA) and small amounts of CLA (0.4 g/100 g total FA) [1].

### 2.10. Milk from Reindeer and Elk 

Reindeer and elk are known for their dense milk, with a creamy consistency and with very high levels of fats and proteins.

In reindeer’s milk, the total fat can be more than six times higher, and the protein content is four times higher, than that of CM. The high protein content also implies a high content in amino acids, in quantities that are 2–6 times those present in CM. This aspect suggested its possible use as a protein supplement, especially for athletes. About 80% of reindeer milk proteins are represented by casein (similar to CM in this sense). The FA profile of reindeer milk is similar to that of CM: the predominant FA are C16: 0, C18: 1, C18: 0 and C14: 0; also, it contains 3 g of trans fatty acids/100 g of total FA and 2 g of LA/100 g of total FA.

As for elk’s milk, no information has been found on the protein profile and very few on the lipid profile: the quantity of saturated fats is lower than that of CM, while that of PUFA is higher. It also has more linoleic acid than reindeer milk.

Both milk alternatives have low levels of lactose (about 50% of the value of CM), have a high mineral salt content, and high values of calcium, sodium, and phosphorus have been reported in elk milk [1].

### 2.11. Mammalians’ Milk Micronutrients 

Table 1 reports the macro- and micronutrient compositions of the different types of mammalian milk alternatives. In particular, by comparing the different types of milk, it can be noted that common elements are iron deficiency, sodium, and good calcium intake. Elk’s milk contains significant amounts of selenium, while the milk of buffalo, goat, sheep, and camel are good sources of vitamin A. Sheep’s milk is rich in riboflavin while the cow’s, goat’s, buffalo and camel’s milk have lower, although adequate, sources. Buffalo’s milk has a high content of vitamin B6 and has good biotin content. Milk of sheep, mare, and dromedary can be considered sources of vitamin C, containing respectively an average of 4.6, 4.3 and 3.8 mg/100 g; the milk of camel also has a higher content of vitamin D [1].

Box 1 **Key Points: Mammalian Milk Alternatives.**

- Buffalo’s milk has higher amounts of fat and therefore gives more energy than cow’s milk (CM). The protein level is slightly higher than that of CM, while the amount of lactose is comparable.
- Goat’s milk has quantities of lipids and proteins similar to CM. It is deficient of vitamin B12 and folate.
- Sheep’s milk has higher amounts of lipids, proteins, and lactose than CM.
- Horses’ milk (donkey, mare) has similar quantities of protein and lactose as that of breast milk, but a lower concentration of lipid (qualitatively it contains, however, more polyunsaturated fatty acids (PUFA) than saturated). Mare’s milk has a higher content of vitamin C.
- Milk of dromedary and camel have a composition similar to CM. Concerning proteins’ content, β-lactoglobulin levels are not measurable (similar to breast milk); the most common serum protein is α-lactalbumin; the main casein is β-casein (similar to breast milk). Camel’s milk also has a higher vitamin D content.
- Yak’s milk has a composition similar to buffalo’s milk. Compared to CM, yak’s milk has an almost double quantity of β-lactoglobulin, and 2 to 6 times higher levels of lactoferrin.
- Reindeer and elk milk have higher amounts of proteins and lipids and less lactose than CM (about 50% less).
- In conclusion, these mammalian milk alternatives are not suitable for infant nutrition.

## 3. Cow’s Milk Protein Allergy: Special Formulas and Milk Alternatives 

CM is the most frequently encountered dietary allergen in infancy. The long-term prognosis for the majority of infants with CMPA is favorable, with 80–90% naturally acquiring tolerance to CM by the age of 6 years. The estimated prevalence of CMPA in the first year of life is 1.5–3% and decreases to less than 1% in children aged six years and to an incidence of 0.1–0.5% into adulthood [4,5]. 

The current treatment of CMPA relies on strict elimination of milk and dairy from the diet and emergency treatment in case of reactions from accidental exposure [6]. The elimination of CM and dairy from the diet of a child carries increased nutritional risk, as these foods represent the main source of protein, fat, calcium, phosphorus and vitamin B12 for infants [7,8]. Maternal breastfeeding is the best option if CMPA occurs during this period, as it is considered the optimal source of infant nutrition [9,10,11]. Mothers should be encouraged to breastfeed and do not require dietary dairy restrictions unless symptoms while breastfeeding [11].

However, when breastfeeding is either not possible or insufficient, replacement with a suitable hypoallergenic milk-formula is mandatory in children less than two years of age [11,12,13] (Table 2). 

### 3.1. Extensively Hydrolyzed Cow’s Milk Formula

An extensively hydrolyzed CM formula (EHF) is the result of a complex manufacturing process using extensive enzymatic hydrolysis and ultra-filtration CM proteins (i.e., whey protein or casein). Currently, EHFs are recommended as the formulas of choice for the treatment of CMPA in the vast majority of cases [11,12,13]. 

EHFs are nutritionally adequate and well tolerated. The main drawbacks of EHFs are a bitter taste and high financial cost (two to three times higher than standard milk formulae) [14]. Notably, in 5–10% of cases, EHFs can potentially cause allergic reactions, due to the presence of short, specific peptides sequences, with potential immunogenic capacities. For this reason, EHFs are not recommended for infants with either a history of anaphylaxis or allergic reaction to CM while exclusively breastfed, who should instead receive an amino acid formula (AAF) [11,12,13].

### 3.2. Amino Acid-Based Formula

AAFs provide proteins in the form of free amino acids and therefore are considered the only completely non-allergenic milk formulas. AAFs would be suitable first-line formulas for all children with CMPA, but are usually reserved, due to their higher cost and low palatability [14], for those infants with one of the following conditions:

- lack of response or reacting to EHF;
- allergic symptoms while exclusively breastfed;
- faltering growth, in particular with multisystem involvement (gastrointestinal tract and/or skin) and multiple food allergies/eliminations;
- severe symptoms, such as anaphylaxis [11,12,13].

In addition, an AAF can be indicated in children with gastrointestinal non-IgE-mediated food hypersensitivities such as:

- eosinophilic esophagitis;
- eosinophilic enteropathies;
- severe forms of food protein-induced enterocolitis syndrome (FPIES) [11,12,13].

### 3.3. Soy-Based Formula

Soy-based formulas are well tolerated in infants with CMPA. Many nutritional deficiencies with these formulas have been reported in the past. Current soy formulas are supplemented with appropriate quantities of amino acids such as methionine, taurine, and carnitine. They are not deficient in iron, zinc, calcium, phosphorus [11]. The content of aluminum is more than 50 times greater in soy formulas than in breastmilk, but this is even truer for soy hydrolyzed formulas (80 times greater). However, 95% of the ingested aluminum is not absorbed in the gut, and the kidney excretes the absorbed 5%, so there are no differences in plasma aluminum levels in children fed with different formulas [11]. Similar considerations are valid for manganese. Soy formulae used to contain phytates which were blamed for their chelating capacity, preventing the proper absorption of micronutrients. Today, however, phytates are almost totally removed from the soy formulae. Two potential issues remain for the use of soy formulas. One is the concern about possible hormonal effects on the reproductive system presumed due to isoflavones present in soy protein. To date, the data do not support those concerns [15]. The other problem to take into consideration is the use of transgenic soy in formulas. The US Department of Agriculture records that up to 93% of soybean crops are transgenic. Due to these nutritional disadvantages, higher allergenicity and less tolerance, the European Academy of Allergy and Clinical Immunology (EAACI) and the European Society for Paediatric Gastroenterology Hepatology and Nutrition (ESPGHAN) recommend not giving soy to infants with CMPA during the first 6 months of life and to children who have experienced gastrointestinal symptoms [11,12,13].

### 3.4. Rice-Based Hydrolyzed Formula

Rice is one of the less allergenic foods, reacting in less than 1% of allergic children. It has no lactose and no phytoestrogens. For this reason, hypo-allergenic formulae containing hydrolyzed rice proteins have been developed. These formulae have now been in use for more than a decade in several westernized countries. Rice protein composition is naturally different from bovine proteins: although they are rich in essential amino acids, three of these do not reach the respective value contained in breastmilk.

For this reason, to guarantee nutritional safety to infants allergic to CM or soy, partially hydrolyzed rice proteins formulas (HRF) are supplemented with lysine, threonine, tryptophan, carnitine and taurine, iron and zinc [11]. Although several studies have shown the HRF to be nutritional and allergy safe, they are still recommended as a second choice [16]. They can be effective in patients who find EHF unpleasant or not tolerated and in subgroups of infants with severe forms of CMPA. Concerning the issue related to Arsenic in commonly-used HRF, the available evidence suggests inorganic arsenic levels within European Food Safety Authority (EFSA)/World Health Organization (WHO) limits in HRF commercialized in Italy, France, and Belgium [17].

### 3.5. Supplementation with Calcium and Vitamin D in Individuals with Cow’s Milk Allergy on Elimination Diet 

In infants with a diagnosis of CMPA, the daily intake of calcium and vitamin D should be regularly assessed to guarantee appropriate quantities. The recommended dietary intake (RDI) for calcium and vitamin D are 200 mg and 400 international units (IU) (10 µg) daily respectively in the first six months of life; 260mg and 400 IU in infants between six and 12 months; and 700 mg and 600 IU between the first and the third year of life [18]. The majority of special formulae are supplemented with calcium and vitamin D in variable quantities. It is necessary to assess from time to time calcium and vitamin D content in the amount of given milk and the remaining infant’s diet. In case the consumption of the special formula is less than 500 mL/die supplementation with 500 mg/die of calcium is required [19]; 400 IU of vitamin D should be administered from birth to the first year of life. Afterward, it is appropriate to continue the supplementation with 600 IU/die.

### 3.6. Unsuitable Mammalian Milk Substitutes in Children with Cow’s Milk Allergy

In infants with a diagnosis of CMPA, low palatability and high cost of recommended hypoallergenic milk formulae are increasingly influencing parents towards frequently choosing as alternative other mammalian milk types. However, these milk alternatives are not always safe or nutritionally adequate. Some studies have suggested that goat’s milk is less allergenic than CM. Although containing the same proteins of CM, some of these in goat’s milk differ in their genetic polymorphisms resulting in less allergenic potential [7]. However, several studies have demonstrated that goat’s milk is unsuitable for children with CMPA because of the cross-reactivity between casein proteins contained in CM and goat’s milk. Therefore, international guidelines recommend not to use goat’s milk as a substitute for CMPA [20]. Similar recommendations also apply to sheep’s milk due to the risk of cross-reactivity with CM [21]. Homologies in amino acidic composition could justify the cross-reactivity observed between proteins from different animal species. On the other hand, the phylogenetic difference could be responsible for the failed recognition of camel proteins by circulating IgE and monoclonal antibodies [22]. Moreover, a recent study showed that camel and cow’s milk have a low cross-reactivity, indicating a low protein similarity. Results demonstrate that camel milk, nutritionally modified, could be a promising alternative to CM-based hypoallergenic infant formulas [23].

Box 2 **Key Points: Cow’s Milk Protein Allergy: Special Formulas and Milk Alternatives.**

- Casein or whey protein-based extensively hydrolyzed formulas (EHFs) are recommended as the formula of choice in infants with *cow’s milk protein allergy* (CMPA), with no history of anaphylaxis or symptoms while exclusively breastfed.
- Soy formula can be used as a second choice in infants with CMPA older than six months of age and with no gastrointestinal symptoms.
- Rice-based hydrolysate formula can be used as the second choice in infants with CMPA who refuse an EHF.
- In children with IgE-mediated CMPA at high risk for anaphylactic reactions (prior history of anaphylaxis and currently not using an EHF), an amino acid formula (AAF) should be suggested rather than an EHF.
- Goat and sheep’s milk are unsuitable milk substitutes for infants with CMPA.
- Regarding milk with a low cross-reactivity (i.e., equine’s, mare’s and donkey’s milk), they are unsuitable during the first year of life in infants with CMPA if not “nutritionally modified.” Even if modified, each cow’s milk substitute should be nutritionally adequate and tested in clinical trials.

## 4. Plant-Based Beverages

Over the last decade, CM consumption pro capita has progressively decreased along with increased availability and consumption of plant-based beverages [24]. Non-dairy beverages continue to show an increasing sales trend in westernized counties because foods labeled as natural are perceived to be the most healthy and appropriate nutritional choice by most consumers [25,26]. 

These beverages are liquid-based extracts of legumes, oilseeds, cereals, and pseudocereals that simulate CM appearance and consistency [27]. However, according to 1308/2013 regulation, it is not possible to use the term “milk” for plant-based drinks. Only what is obtained by milking can be called “milk”; so, except for almond and coconut milk, all the other products can be named as “beverage” or “drink.” Concerning the nutritional proprieties, these drink features are different from those related to the common CM or breast milk. It is as yet unclear if their consumption may be associated with any beneficial effect on health but it is well known that an inadequate substitution of formulas or CM (after the first year of life) with vegetable drinks can be related to major nutritional gaps and Kwashiorkor in younger children, especially if the plant-based drink is the only, or predominant child diet [25,26,27,28]. The main reasons that over time have affected consumers’ choice increasingly towards vegetable drinks are different: medical reasons (lactose intolerance and CMPA), hypercholesterolemia, more preference to vegan diets, unfounded concerns regarding antibiotics and growth hormones residues in CM and sense of a healthier choice; for these reasons it has been estimated that 15% of the European population avoids dairy products [27]. According to the definition, vegetable milk replacements are colloidal suspensions or emulsions including dissolved and disintegrated vegetable material: these are traditionally prepared by milling different raw material in suspension and then by filtering it to remove bigger particles [27]. Plant-based beverages can be classified in five categories: cereal-based (oat, rice, corn, spelled); legumes-based (soy, peanut, lupin, cowpea); nut-based (almond, coconut, hazelnut, sunflower); pseudocereals-based (quinoa, teff, amaranth).

### 4.1. Soy Drinks

The use of soy milk was first reported about 2000 years ago in China. Soy milk was the first plant-based milk which served the purpose of providing nutrients to a population where the milk supply was inadequate [25]. Soy beverage contains much lower carbohydrates and fats compared to CM. Therefore, it has a lesser energy value, while the protein supply is also lower. Regarding the lipidic profile, it contains low levels of saturates, while it represents a good source of trans fats, MUFA, and PUFA (ALA and LA).

Regarding micronutrients, it contains isoflavones probably responsible for the beneficial effects of soy against cancer, cardiovascular disease, and osteoporosis; phytosterols widely recognized for their cholesterol-lowering properties [25]. Soy drink shows calcium and vitamin B-12 deficit; for this reason, those micronutrients are often supplemented. The claimed benefits of consuming soy milk include the absence of lactose and cholesterol, high nutritive value, higher protein quality compared to other beverages, and high digestibility. However, these drinks should not be given to younger children (early years of life). In this regard, it is judged necessary to resort to a 3 or 7 days food diary to define the micronutrient intake according to age needs. Unfortunately, a well-known disadvantage of soy milk preparation is a characteristic beany flavor [27]. Furthermore, soy beverage cannot be used in individuals allergic to soy proteins as it may result in possible flatulence.

### 4.2. Almond Milk

Almond milk, compared to bovine milk, has less protein content while the amount of carbohydrates and fats almost compares to those in CM. Regarding the lipidic profile, it presents fewer levels of saturates and higher levels of trans-fats, MUFA (oleic acid) and PUFA (ALA and LA). Regarding almond milk micronutrients, it has good levels of vitamin E, an important antioxidant, and manganese. Almonds are also a rich source of other nutrients such as calcium, potassium, magnesium, iron, selenium, copper, and zinc [19]. This nutritional profile makes it unsuitable as the only food in a baby’s diet. If given as a milk substitute, it would be essential for fortification by adding critical micronutrients like calcium and B12, based on the growing need. The stated benefits of almond milk are the cholesterol-lowering power and potential prebiotic features, which may determine the bifidobacteria growth [25]. Generally, this drink is nutritionally better than other plant-based beverages, and it represents a good trans-fat and vitamin E source but has downsides too: the prevalence of nut allergies and high price limit the consumption [25]. Despite its characteristics, it cannot be considered as a milk substitute but as a beverage to be given to children during snack time.

### 4.3. Rice Drink

The rice drink is rich in simple sugars, so energy is readily available. It has a lower level of fats compared to other beverages: it does not contain saturated but mainly unsaturated MUFA and PUFA.

Furthermore, regarding the protein content, the rice drink has the lowest amount of protein compared to the other plant-based drinks. The micronutrients, calcium, magnesium, and iron levels are comparable to CM, while the rice drink has more vitamin A and E. A brief mention has to be made regarding the incorrect use of this drink in infants with CMPA, as parents are often not aware of the fundamental difference between the rice-based formula and the beverage, which can have significant effects on growth and development. Furthermore, high levels of arsenic have been detected in rice drinks used by children as recently pointed out by the European Society for Paediatric Gastroenterology Hepatology and Nutrition (ESPGHAN) Nutrition Committee (not in hydrolyzed rice-based formulas), the reason why it is recommended avoiding rice-based drinks consumption in babies and younger children [29].

### 4.4. Coconut Milk

Coconut milk plays an important role in South East Asia cuisine, both as a drink and ingredient used in different recipes. It is, indeed, used quite often as a thickener to make a full-bodied dish [27]. This “milk” has a high content of fat, so it is a high energy drink: in particular, the lipidic profile is characterized by high levels of saturated, especially the hyaluronic acid c, and by low levels of trans fats (this limits the use of this milk). As a counterpart, there is a minor protein content of carbohydrates and fiber.

Regarding micronutrients, there are high levels of potassium, magnesium, iron, and zinc, with a good amount of vitamin E and C. Regarding the hyaluronic acid, being a saturated FA, it is important to monitor the trans fats daily consumption. In more detail, this saturated FA is present in breast milk and has been related to cerebral growth, immune system stimulation, and vessel elasticity upkeep [27]. It has to be mentioned that the beverages on the market usually have a little amount of coconut milk, being extremely diluted and with simple added sugars, which means a considerably lower lipidic profile than the “real” coconut milk.

### 4.5. Oat Drink

Oat drink contains fewer fats, especially PUFA, and proteins, but a better amino acid profile than other beverages. This cereal gains great attention for the presence of fiber, phytochemicals (antioxidants and polyphenols), and its high nutritional level. Beta-glucan, a soluble fiber with the ability to increase the solution viscosity, can delay gastric emptying time, which increases gastrointestinal transit time, thereby reducing blood glucose and cholesterol (total cholesterol and LDL) levels [27]. On the other hand, oat contains a significant amount of phytic acid, an antinutrient (which interferes with some nutrients uptake) and lack of calcium, for which this beverage needs to be fortified.

### 4.6. Plant-Based Beverages versus Cow’s Milk

It is crucial to focus on the differences between the essential features of plant-based beverages compared to CM (Table 3). Regarding the protein content, only the soy milk values are similar to the CM ones, with a protein content that goes from 2.9% to 3.7%; all the other beverages show deficient protein levels, but the quinoa, hemp, and oat drinks are the only ones showing a content >1%. Plant proteins are generally of lower nutritional quality compared to animal-derived proteins due to limiting amino acids (lysine in cereals, methionine in legumes) and poor digestibility [30]. The nutritional inferiority of these beverages represents a risk, especially when given to younger children as CM substitutes, without knowing the peculiar differences [30]. In this regard, in the past few years, there has been an inappropriate consumption of these beverages as alternatives to infant formula, especially in the case of supposed CMPA. It is worth raising some findings: their composition does not follow European guidelines; are low energy drinks, and with proteins, vitamins, and mineral levels inadequate for early childhood. These drinks can lead to a severe nutritional deficit in babies. In fact, between 2008 and 2011, there were nine cases of severe nutritional lack caused by vegetable drink consumption (age between four and 14 months old). The beverages involved in these cases were rice, soy, almond, and chestnut based; three of these nine severe malnutrition cases also showed severe hypoalbuminemia (<20 g/L) and spreading edema [26].

Regarding the lipidic profile, vegetable substitutes generally have low saturated FA levels, except for coconut milk [27]; despite this, some products show good energy producing levels similar to the whole CM, due to sugars and other carbohydrates [25].

Furthermore, some of these beverages contain added sugars and sweeteners, and there is a difference regarding the carbohydrate profile: the absence of lactose and galactose in vegetable drinks [31]. A recent research finding has led to a different glycemic-index (GI) in plant-based beverages pointing out high levels in rice and coconut drinks (GI > 96) due to a high glucose content; oat drink (GI = 59) due to b-glucan content; low levels for different brands of soy drink (GI = 47–61) and almond milk (GI = 49–64). In contrast to the others, the rice-based drink also showed a high glycaemic level [31].

Due to low proteins, vitamins (B12, B2, D, and E) and mineral content (especially calcium) in most of the plant-based drinks, usually they need to be fortified. Nevertheless, fortified plant-based beverages and CM remarkably differ in nutritional properties as some nutrient bioavailability may considerably vary [25].

For all these reasons, CM should not be removed and substituted with these drinks in younger children’s diets, unless in the presence of medical conditions [25]. It is, therefore, mandatory to remember that every “milk” or “drink” has peculiar characteristics with potential benefits and disadvantages.

Box 3 **Key Points: Plant-Based Beverages.**

- All the plant-based drinks should not be used as a substitute for cow’s milk (CM) in children <24 months old. Additionally, some of these beverages contain added sugars and sweeteners.
- Soy drink contains fewer sugars and fats, especially trans fats, compared to CM. It contains isoflavones and phytosterols. It lacks calcium and vitamin B12. It does not contain cholesterol and lactose. It should not be given to children allergic to soy proteins.
- Almond milk is rich in vitamin E and trans fats, and it could be given as drink during snack time.
- Rice drink has fewer lipids (especially polyunsaturated fatty acids) and proteins than CM. It has a higher vitamin A and D content. It contains arsenic, so it is not recommended in babies and younger children.
- Coconut milk has higher amounts of fats, potassium, magnesium, iron, zinc, vitamin C and E and a lower amount of protein, sugars, and fiber compared to bovine milk.
- Oat drink has a lower amount of fats, proteins, and calcium than CM. It contains an antinutrient that hampers some nutrients’ absorption. It has cholesterol-lowering properties.

## 5. Conclusions

CM and dairy products form an integral part of a growing child’s nutritional requirements. The emergence of allergy and intolerances to CM and vegan dietary preferences has lead to an increased need and utilization of CM substitutes, including other mammalian milk types and plant-based milk beverages. However, many of these milk alternatives do not necessarily address the nutritional requirements of infants and children. It is, therefore, important to select the most appropriate option for each patient. Also, it should be noted that unmodified cow’s and other mammalian’s milk types, as well as plant-based beverages, are not recommended for healthy infants in the first year of life, who should receive instead appropriate CM or goat’s milk-based formula [32]. Finally, there is a strong need to promote awareness about qualitative and quantitative nutritional compositions of different milk formulas, in order to guide medical providers selecting the best option for children with CMPA.

## Figures and Tables

**Table 1:** Nutritional composition of different types of milk of animal origin. Source: Food and Agriculture Organisation of the United Nations (FAO) [1].

Composition in 100 g																
	Human	Cow	Buffal	Goat	Sheep	Yak	Mare	Donkey	Dromedary	Camel	Mithun	Musk ox	Llama	Alpaca	Reindeer	Elk
Energy (kcal)	70	62	99	66	100	100	48	37	56	76	122	85	78	71	196	129
Water (g)	87.5	87.7	83.2	87.7	82.1	82.6	89.8	90.8	89	84.8	78.6	83.6	84.8	83.7	67.9	76.8
Total protein (g)	1.0	3.3	4	3.4	5.6	5.2	2	1.6	3.1	3.9	6.5	5.3	4.1	5.8	10.4	10.5
Total fat (g)	4.4	3.3	7.5	3.9	6.4	6.8	1.6	0.7	3.2	5	8.9	5.4	4.2	3.2	16.1	8.6
Lactose	6.9	4.7	4.4	4.4	5.1	4.8	6.6	6.4	4.3	4.2	4.4	4.1	6.3	5.1	2.9	2.6
Ash	0.2	0.7	0.8	0.8	0.9	0.8	0.4	0.4	0.8	0.9	0.9	0.16	0.7	1.6	1.5	1.6
**Minerals**																
Calcium (mg)	32	112	191	118	190	129	95	91	114	154	88		195		320	280
Iron (mg)		0.1	0.2	0.3	0.1	0.6	0.1		0.2							0.3
Magnesium (mg)	3	11	12	14	18	10	7	4	13	8			15		19	23
Phosphorus (mg)	14	91	185	100	144	106	58	61	86	132	147		122		270	276
Potassium (mg)	51	145	112	202	148	95	51	50	151	186			120		156	111
Sodium (mg)	17	42	47	44	39	29	16	22	66	66			27		48	78
Zinc (mg)	0.2	0.4	0.5	0.3	0.6	0.9	0.2	0	0.6	0.7					1.1	0.6
Copper (mg)	0.1				0.1	0.1	0.1	0	0.2							0.3
Selenium (μg)	1.8	1.8		1.1	1.7											11
Manganese (μg)		8		18	18				106							1
**Vitamins**																
Retinol (μg)	60	35	69	45	64											
Carotene (μg)	7	16		13												
Vitamin A (μg RE)	61	37	69	48	64					97						
Vitamin E (mg)	0.08	0.08	0.19	0.05	0.11					0.15						
Thiamine (mg)	0.01	0.04	0.05	0.06	0.07		0.03	0.06		0.01						
Riboflavina (mg)	0.04	0.2	0.11	0.13	0.34		0.02	0.03	0.06	0.12						
Niacin (mg)	0.18	0.13	0.17	0.24	0.41		0.07	0.09								
Panthotenic acid (mg)	0.22	0.43	0.15	0.3	0.43											
Vitamin B6 (mg)		0.04	0.33	0.05	0.07					0.05						
Folate (μg)	5	8.5	0.6	1	6											
Biotin (μg)		2	13	2.5	2.5											
Vitamin B12 (μg)	0.05	0.51	0.4	0.07	0.66											
Vitamin C (mg)	5	1	2.5	1.1	4.6		4.3		3.8	3						
Vitamin D (μg)	0.1	0.2		0.1	0.2					1.6						

**Table 2:** General characteristics of infant formulas for cow’s milk protein allergy (modified from Fiocchi A., et al.; World Allergy Organ J 2016 [11]).

Energy	Similar to Human Milk
Proteins	Within normal recommended ranges, but cow’s milk protein are hydrolysate, or whole proteins different than human milk proteins; some supplemented with lysine, threonine or tryptophan
Fats	Only 15% have α-linolenic acid in similar amounts to human milk; 31% have more linoleic acid than human milk; 46% do not include docosahexaenoic acid (DHA); one includes 25% palmitic acid in β position
Carbohydrates	70% of special formulae are without lactose; all have a content of carbohydrates higher than human milk
Micronutrients	Iron ≤ than in human milk (risk of iron-deficiency). Content of other minerals should be reviewed considering other factors
Vitamins A, E, D	Need to be reviewed the doses depending on other factors (>25% of children consumed <2/3 of the Recommended dietary intake (RDI) of calcium, vitamins D and E)
Nucleotides	77% have nucleotides
Choline	Big variability in choline levels between different formulae
Taurine	92% have taurine
Carnitine	92% have carnitine
Prebiotics	15% include fructo-oligosaccharides (FOS) and galacto-oligosaccharides (GOS)
Probiotics	8% include probiotic

**Table 3:** Nutritional comparison (per 100 g) between cow’s milk vs. selected plant-based milk alternatives *.

	Whole Cream Cow’s Milk (FAO/IEO)	Soy-Based Beverages (IEO)	Coconut Milk (IEO)	Almond Milk (IEO)	Rice-Based Beverages (USDA)
Energy, kcal	62	32	236	56	47
Total Proteins (g)	3.3	2.9	2.3	1.3	0.28
Total Fats (g)	3.3	1.9	23.8	3.3	0.97
Cholesterol (mg)	11	0	0	0	0
Available Carbohydrates (g)	4.7	0.8	3.3	5.5	9.17
Total Dietary Fibres (g)	0	0	2.2	0.8	0.3
Water (g)	87.8	89.7	67.6	89.2	89.28
*Fatty Acids*					
Total Saturates (g)	2.11	0.21	21.14	0.28	0
Lauric Acid, (g)	0.11		10.58	0	
Myristic Acid, (g)	0.37		4.18	0	
Palmitic Acid, (g)	0.92		2.02	0.21	
Stearic Acid, (g)	0.39		1.23	0.06	
Total Monounsaturates (g)	1.1	0.33	1.01	2.37	0.625
Oleic Acid (g)	0.93	0.32	1.01	2.34	
Total Polyunsaturates (g)	0.12	0.83	0.26	0.65	0.313
Linoleic Acid (g)	0.07	0.73	0.26	0.63	
Linolenic Acid (g)	0.05	0.1	0	0.02	
*Micronutrients*					
Calcium (mg)	112	13	16	14	118
Sodium (mg)	42	32	15	1	39
Potassium (mg)	145	120	263	47	27
Magnesium (mg)	11		37	16	11
Iron (mg)	0.1	0.4	1.6	0.2	0.2
Zinc (mg)	0.4	0.2	0.67	0.16	0.13
Vitamin A (µg)	37	1	0	0	63
β-carotene (µg)	16		0	0	
Total Folates (µg)	8.5	19	16	3	2
Vitamin B12 (µg)	0.5		0	0	0.63
Vitamin B6 (µg)	0.04	0.07	0.03	0.1	0.04

* Reference: European Institute of Oncology (IEO); Food Composition Database for Epidemiological Studies in Italy (BDA); Food and Agriculture Organisation of the United Nations (FAO); U.S. Department of Agriculture (USDA).
